# Dermoscopy and Trichoscopy in Dermatomyositis—A Cross-Sectional Study

**DOI:** 10.3390/jcm11020375

**Published:** 2022-01-13

**Authors:** Magdalena Żychowska, Adam Reich

**Affiliations:** Department of Dermatology, Institute of Medical Sciences, Medical College of Rzeszow University, 35-959 Rzeszow, Poland; adi_medicalis@go2.pl

**Keywords:** dermoscopy, videodermoscopy, trichoscopy, capillaroscopy, Gottron’s sign, Gottron’s papules, dermatomyositis

## Abstract

Background: (Video)dermoscopy is a non-invasive diagnostic technique that has a well-established role in dermatooncology. In recent years, this method has also been increasingly used in the assessment of inflammatory dermatoses. So far, little is known about the (video)dermoscopic features of dermatomyositis (DM). Methods: Consecutive patients with DM were included in the study and videodermoscopic assessments of the nailfolds, scalp, and active skin lesions were performed. Results: Fifteen patients with DM (10 women and 5 men) were included. Capillaroscopy showed elongated capillaries (90.9%), avascular areas (81.8%), disorganized vessel architecture (81.8%), tortuous capillaries (72.7%), dilated capillaries (72.7%), and hemorrhages (72.7%). The trichoscopic findings included linear branched vessels (80.0%), linear vessels (60.0%), linear curved vessels (53.3%), perifollicular pigmentation (40.0%), perifollicular erythema (33.3%), scaling (20.0%), white (20.0%) or yellow (20%) interfollicular scales, and white (20.0%) or pinkish (13.3%) structureless areas. Polymorphic vessels of an unspecific distribution and white or pink structureless areas were frequently observed under dermoscopy in cutaneous manifestations of DM, including Gottron’s papules and Gottron’s sign. Conclusions: Dermoscopy of the nailfolds (capillaroscopy), scalp (tricoscopy), and active cutaneous lesions may be of value in the preliminary diagnosis of DM.

## 1. Introduction

Dermatomyositis (DM) is an autoimmune disorder from the spectrum of inflammatory myopathies. The disease may present with various skin manifestations and, therefore, it might pose a diagnostic challenge [1]. Skin involvement may precede the development of myositis. Therefore, early diagnosis may be crucial for prompt management of the patients.

Gottron’s papules (violaceous papules on dorsal hands over interphalangeal and metacarpophalangeal joints) and heliotrope (violaceous erythema, commonly associated with oedema, and predominantly affecting upper eyelids) are considered to be pathognomonic for DM. Another common skin symptom is Gottron’s sign, consisting of violaceous macules over joints, especially over knees and elbows. The presence of erythematous lesions on the lateral aspects of the thighs is referred to as a “holster sign”. Photosensitivity is frequently observed in DM and may manifest with erythema in the upper chest (“V-neck sign) and upper back (“shawl sign”). However, in the case of severe photosensitivity, the skin involvement may be much more extensive. Nailfold lesions are a frequent finding and include periungual teleangiectasia, dystrophic cuticle, and small hemorrhagic infarcts. Other DM skin lesions on the hands include fissures and hyperkeratosis on the fingers and palms, which are referred to as “mechanic’s hands” [2].

Scalp lesions in DM may present as erythema owing to photosensitivity or atrophic and erythematous-scaly lesions frequently accompanied by pruritus. Non-scarring alopecia may also occur.

Other cutaneous manifestations of DM include but are not limited to calcinosis, flagellate erythema on the trunk, erythroderma, inverse Gottron’s papules (white triangular hyperkeratosis on the palmar surface of interphalangeal joints), and hyperkeratotic papules on the extensor surfaces of the extremities (Wong variant of DM) [3,4].

A skin biopsy may be useful in differentiating DM from other dermatoses, but it is not sufficient to differentiate DM from lupus erythematosus. Still, it is an invasive procedure, associated with the risk of scarring. In addition, characteristic histopathological changes are observed in classical skin manifestation such as Gottron’s papules, but are not present in unspecific lesions such as “mechanic’s hands” or follicular hyperkeratosis [3,4].

(Video)dermoscopy is a non-invasive diagnostic technique that has a well-established role in the early diagnosis of melanoma and non-melanoma skin cancer. In recent years, this method has also been increasingly used in the assessment of inflammatory dermatoses [5,6]. So far, only a few reports have been published on the dermoscopic features of DM [7,8,9,10,11,12,13,14,15].

The aim of the current study was to analyze videodermoscopic features of various skin manifestations of DM.

## 2. Materials and Methods

This prospective cross-sectional study was conducted in the Department of Dermatology in Rzeszow, Poland, from November 2020 to October 2021. Patients who fulfilled the Bohan and Peter criteria and/or the European League Against Rheumatism/American College of Rheumatology (EULAR/ACR) IIM criteria were recruited to the study. The exclusion criteria were as follows: absence of active cutaneous manifestations of DM, presence of relevant concomitant dermatological conditions that might affect the dermoscopic assessment, overlap syndromes with other connective tissue diseases, and lack of a definite diagnosis of DM. The participants underwent a full physical examination. Dermoscopic assessments were performed using Canfield D200^EVO^ Videodermatoscope (Canfield Scientific GmbH, Bielefeld, Germany) at 20–70-fold magnification. In the first step, the examination was performed without immersion fluid (“dry videodermoscopy”) in order to better assess the scales. In the second step, videodermoscopy with ultrasound gel and minimal pressure (“wet videodermoscopy”) was conducted to obtain better visualization of the vessel morphology. The dermoscopic examinations were performed before the treatment initiation or, if the patient was on maintenance therapy and experienced a flare of DM, before any significant modification of the treatment.

Dermoscopy of the nailfolds (capillaroscopy) and scalp (trichoscopy) was performed in all patients. In addition, other skin lesions present in individual patients were evaluated using dermoscopy.

Videodermoscopy of the nailfolds (capillaroscopy) was performed after acclimatization of each patient for at least 20 min at room temperature. Capillaroscopic photographs of each finger were obtained. Parameters in the analysis of capillaroscopic images included the presence of tortuous capillaries (meandering or curled capillaries), “bushy” capillaries (branched capillaries resembling the morphology of a bush/tree), elongated capillaries, dilated capillaries, giant capillaries, prominent subpapillary plexus (capillary network at the base of the finger), bleedings, disorganized capillaries (irregular pattern), and avascular areas (areas where at least two adjacent capillaries are missing).

Trichoscopic photographs of the scalp were taken, including the frontal, parietal, temporal, and occipital region. In the analysis of scalp images, the dermoscopic features of follicular openings (white dots, yellow dots, black dots, red follicular dots, keratotic plugs, and absence of follicular openings), hair shafts (hair diameter diversity, broken hair, pili torti, circular hair, and absence of vellus hair), perifollicular surface (scaling, erythema, pigmentation, tubular hair casts, and white perifollicular halo), interfollicular surface (structureless areas, scales, pigmentation, honeycomb pigment pattern, blue-grey dots, rosettes, bleedings, globules, and erosions), and vessel morphology (dotted, linear, linear branched, linear curved, and polymorphous vessels) were taken into consideration.

Standardized dermoscopic parameters for the evaluation of non-neoplastic dermatoses were used in the analysis of dermoscopic images of cutaneous lesions in the course of DM. These parameters were developed by an expert consensus on behalf of the International Dermoscopy Society [16], and included the following: vessel morphology (dotted, linear, linear with branches, and linear curved), vessel distribution (uniform, clustered, peripheral, reticular, and unspecific), color of scales (white, yellow, and brown), distribution of scales (diffuse, central, peripheral, and patchy), follicular findings (follicular plugs, follicular red dots, perifollicular white color, and perifollicular pigmentation), presence of other structures (colors or morphologies including structureless areas, dots/globules, and lines and circles), and presence of specific clues. On the basis of personal experience, several additional dermoscopic and trichoscopic features were included in the analysis.

In the dermoscopic and trichoscopic assessments, in line with the previously proposed consensus, pinpoint, globular, and glomerular vessels were classified as dotted vessels; “bushy” vessels as linear vessels with branches; and tortuous vessels as linear curved vessels. However, the terms of “bushy” capillaries and tortuous capillaries were kept in the descriptions of capillaroscopic findings as they are still in common use.

All dermoscopic examinations were performed by the same investigator (M.Z.). Evaluation was performed in an independent manner by two dermatologists with experience in dermoscopy, and discrepancies were discussed until a consensus was reached.

The study was approved by the Bioethics Committee of University of Rzeszów (No. 6/11/2020). Written informed consent was obtained from all study participants.

Data were analyzed using Statistica^®^ 13.0 Software for Windows Software (Statsoft Polska, Kraków, Poland). Categorical data are expressed as absolute numbers and percentages, and continuous data as means with standard deviation (SD) of the mean.

## 3. Results

Fifteen patients with DM (10 women and 5 men; mean age 48.7 ± standard deviation (SD) 19.4 years) were included in the study.

The clinical characteristics of study participants are summarized in Table 1. Capillaroscopic, trichoscopic, and dermoscopic findings in each patient are presented in Table 2, Table 3 and Table 4. To increase the clarity of data presentation, dermoscopic/trichoscopic parameters that were not observed in any of the participants are not included in Table 2, Table 3 and Table 4.

### 3.1. Capillaroscopy

**Figure 1:**
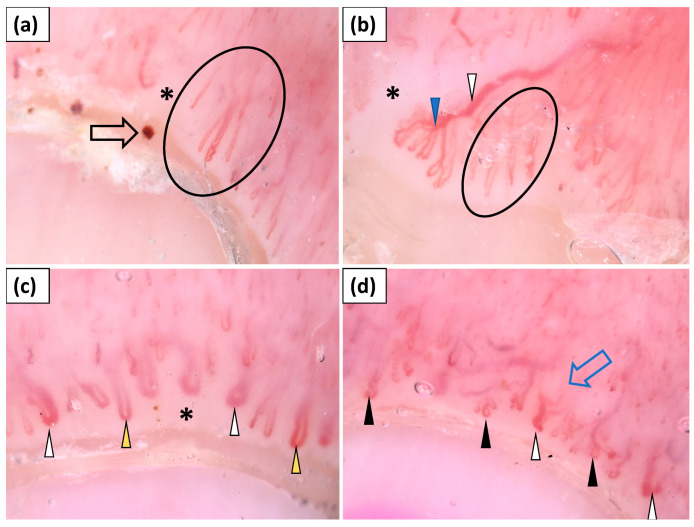
(**a**–**d**) Videodermoscopy of nailfold capillaries showed the presence of elongated capillaries (black circle), tortuous vessels (black arrowhead), “bushy capillaries” (blue arrowhead), dilated vessels (yellow arrowhead), giant capillaries (white arrowhead), hemorrhages (black arrow), avascular areas (black asterisk), and disorganized vessel architecture (blue arrow).

Capillaroscopy (dermoscopy of the nailfolds) was performed in all patients (n = 15). Four patients did not show any abnormalities in nailfold capillaroscopy. In the remaining group, the most frequent findings were elongated capillaries (90.9%), followed by avascular areas (81.8%), disorganized vessel architecture (81.8%), tortuous capillaries (72.7%), dilated capillaries (72.7%), and hemorrhages (72.7%). “Bushy” capillaries and giant capillaries were observed less frequently, in 45.5% and 36.4% of cases, respectively (Table 2, Figure 1).

### 3.2. Trichoscopy

**Figure 2:**
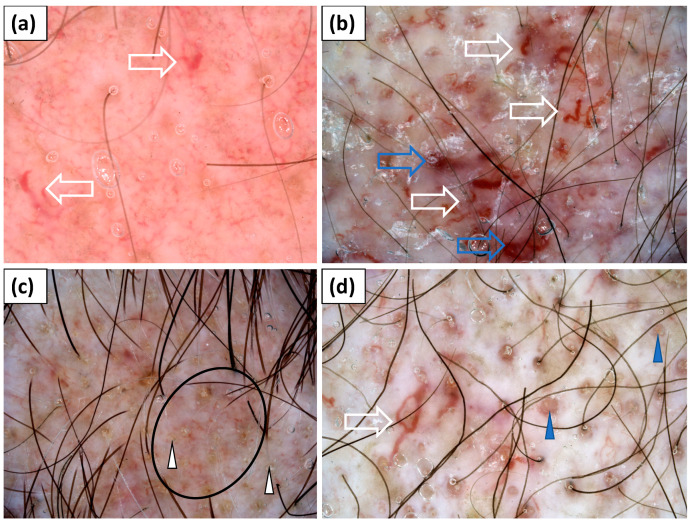
(**a**–**d**) Trichoscopy showed yellow dots (white arrowhead); red follicular dots (blue arrowhead); interfollicular honeycomb pigment pattern (black circle); thick linear, linear branched, and linear curved capillaries (white arrow); and lake-like structures (blue arrow).

Trichoscopy (dermoscopy of the scalp) was performed in all study participants (n = 15). Yellow dots (60.0%), hair diameter diversity (60.0%), and inferfollicular honeycomb pigment pattern (46.7%) were frequently observed. However, these findings were most likely associated with coexisting androgenetic alopecia. Other trichoscopic findings included perifollicular pigmentation (40.0%), perifollicular erythema (33.3%), perifollicular scaling (20.0%), white (20.0%) or yellow (20%) interfollicular scales, and white (20.0%) or pinkish (13.3%) structureless areas. Bleedings and erosions were rarely observed (6.7%). The most common vascular morphology was linear branched vessels (80.0%), followed by linear (60.0%) and linear curved (53.3%) vessels. Dotted vessels were noted in 26.7% of cases. Lake-like structures were present in two (13.3%) patients (Table 3, Figure 2).

### 3.3. Dermoscopy of Gottron’s Papules

**Figure 3:**
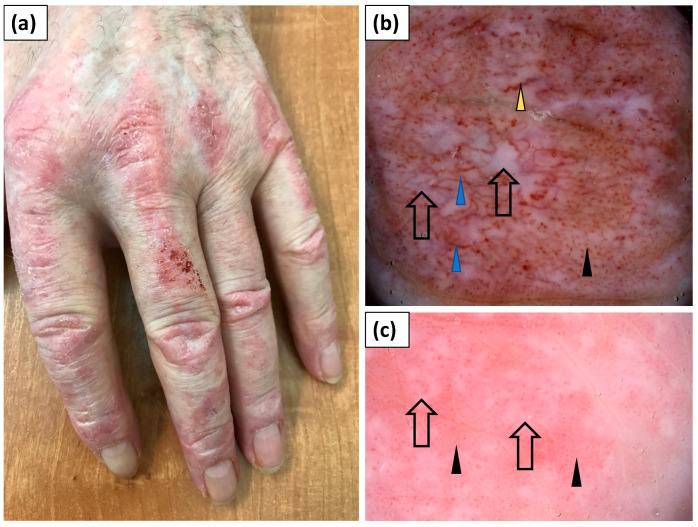
(**a**) Clinical presentation of erythematous papules and plaques over the metacarpophalangeal and interphalangeal joints (Gottron’s papules). (**b**) Videodermoscopy of Gottron’s papules showed irregularly distributed dotted (black arrowhead), linear vessels with branches (blue arrowhead), and linear curved vessels (yellow arrowhead) surrounding white structureless areas (black arrow). (**c**) Videodermoscopy of Gottron’s papules showing predominantly dotted vessels (black arrowhead) surrounding irregular pink structureless areas (black arrow).

Thirteen patients presented with typical papules or plaques on the dorsal aspects of the hands over metacarpophalangeal and interphalangeal joints (Gottron’s papules). The most frequent vessel morphology was dotted vessels, observed in 92.3% of cases. Linear vessels, linear vessels with branches, and linear curved vessels were noted in 53.9%, 46.2%, and 30.8% of cases, respectively. At least two vessel morphologies (i.e., pleomorphic vessels) were present in 76.9% of patients. The vessels were arranged irregularly in an unspecified pattern (100%). White scaling was predominantly (76.9%) observed and showed a patchy arrangement in the majority of cases (69.2%). Pink (76.9%) or white (46.2%) structureless areas were frequent findings. Follicular plugs or red hemorrhagic dots were observed less commonly (each in 38.5% of cases) (Table 4, Figure 3).

### 3.4. Dermoscopy of Gottron’s Sign

**Figure 4:**
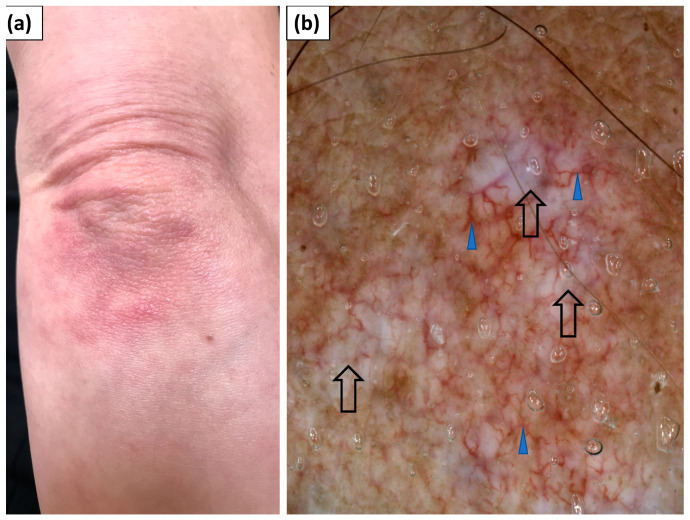
(**a**) Clinical presentation of Gottron’s sign over the elbow. (**b**) Videodermoscopy of Gottron’s sign showed irregularly distributed linear vessels with branches (blue arrowhead) and white structureless areas (black arrow).

**Figure 5:**
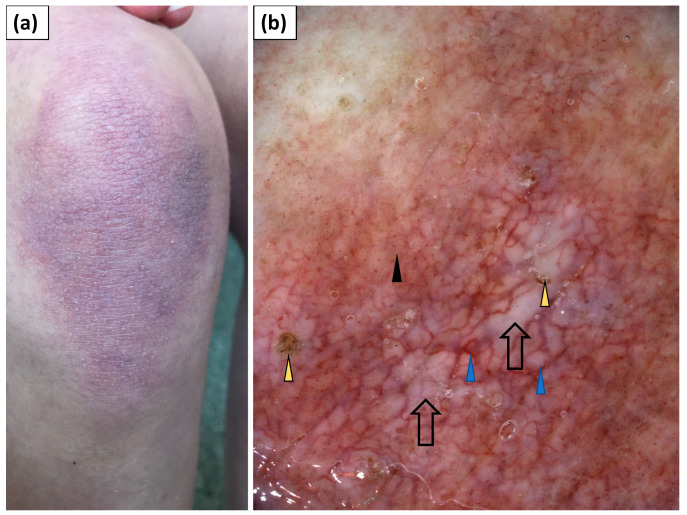
(**a**) Clinical presentation of Gottron’s sign over the knee. (**b**) Videodermoscopy of Gottron’s sign showed irregularly distributed dotted (black arrowhead) and multiple linear vessels with branches (blue arrowhead) surrounding white structureless areas (black arrow). Follicular plugs (yellow arrowhead) were present as well.

In 12 patients, erythematous lesions (Gottron’s sign) were present on the elbows (Figure 4), while they were present in 5 patients on the knees (Figure 5).

Under dermoscopy, Gottron’s sign on the elbows showed dotted (83.3%), linear (66.7%), linear with branches (41.7%), or linear curved (25.0%) vessels. In the majority of patients (83.3%), at least two vessel morphologies (pleomorphic vessels) were present. The vessels showed predominantly an unspecified (75.0%) distribution. White scales were noted in 75% of patients and they were arranged in a patchy pattern in 58.3% of cases. White or pink structureless areas were observed in 41.7% and 33.3% of cases, respectively. In three cases (25%), speckled brown pigmentation was noted (Table 4).

Dermoscopic findings within Gottron’s sign on the knees were largely similar. Dotted vessels were observed in all five (100%) cases and were predominantly accompanied by linear vessels (60.0%) or linear vessels with branches (60.0%). The vessel distribution was unspecified (80.0%) or uniform (20.0%). White scales were noted in 80% of cases and showed patchy (60%) or diffuse (20%) arrangement. Pink and white structureless areas were frequently observed (60% and 40% of cases, respectively) (Table 4).

### 3.5. Dermoscopy of Other Cutaneous Lesions

**Figure 6:**
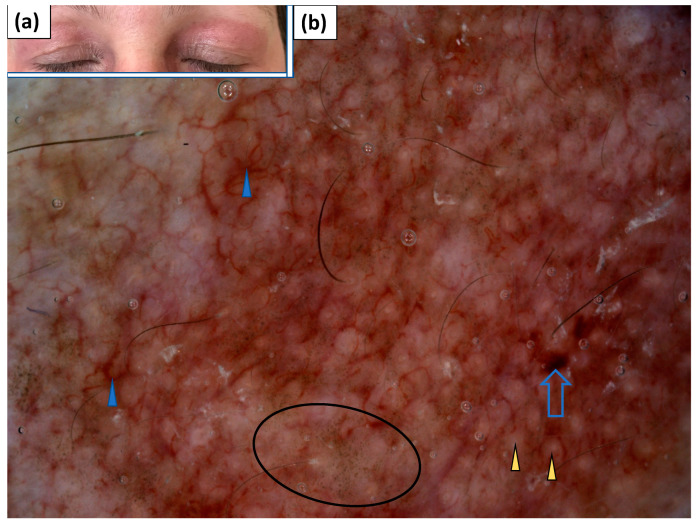
(**a**) Clinical presentation of heliotrope. (**b**) Videodermoscopy of heliotrope showed irregularly distributed linear vessels with branches (blue arrowhead), red hemorrhagic globules (blue arrow), grey dots (black circle), and yellowish globules (yellow arrowhead).

Heliotrope was present in three patients. Linear vessels with branches were noted in all three cases and they showed uniform (66.7%) or patchy (33.3%) distribution. White scales of patchy arrangement were observed in a single case. In one patient, brownish-greyish dots (peppering) were present, and in another patient, red dots were observed under dermoscopy (Table 4, Figure 6).

**Figure 7:**
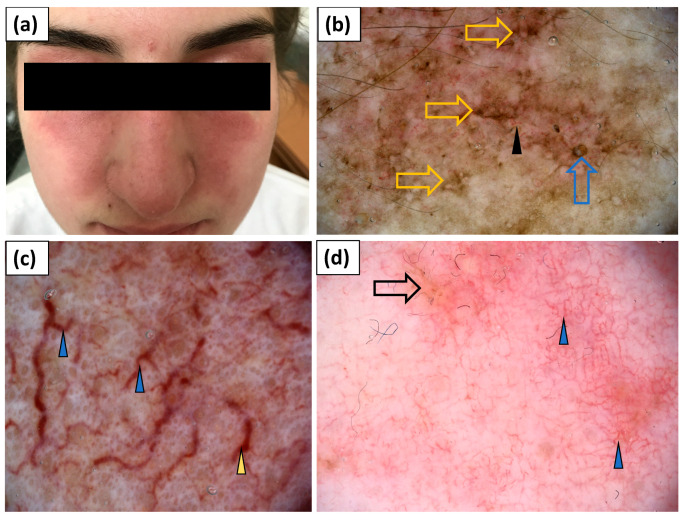
(**a**) Clinical presentation of erythematous lesions on the face. (**b**–**d**) Videodermoscopy of lesions on the face showed irregularly distributed linear vessels (black arrowhead), linear vessels with branches (blue arrowhead) and linear curved vessels (yellow arrowhead), yellow structureless areas (black arrow), follicular plugs (blue arrow), and stellate brown structures (yellow arrow).

Less specific erythematous lesions on the face were noted in seven patients with DM. These lesions showed linear (71.4%), linear with branches (71.4%), or linear curved (42.9%) vessels, of unspecific arrangement in the majority (57.1%) of cases. White (14.3%) or yellow (14.3%) scales were not frequent. Whitish, pink, or yellow structureless areas were noted in single cases (Table 4, Figure 7).

**Figure 8:**
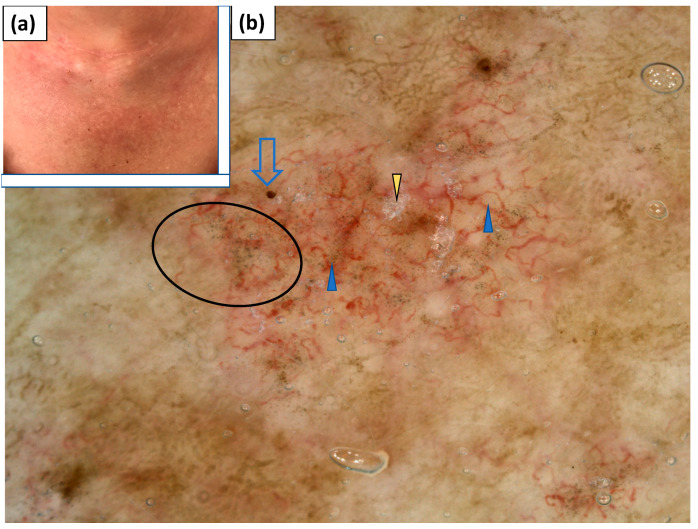
(**a**) Clinical presentation of V-neck sign. (**b**) Videodermoscopy of V-neck sign showed irregularly distributed linear vessels with branches (blue arrowhead), red hemorrhagic globules (blue arrow), grey dots (black circle), and white scaling of patchy distribution (yellow arrowhead).

V-neck sign was observed in two patients. Linear vessels with branches and linear curved vessels were observed in both cases, and were accompanied by dotted and linear vessels in one of the patients. The vessels were arranged in an unspecific pattern. In both patients, white scales of patchy distribution were noted. In one patient, grey dots were observed under dermoscopy (Table 4, Figure 8).

**Figure 9:**
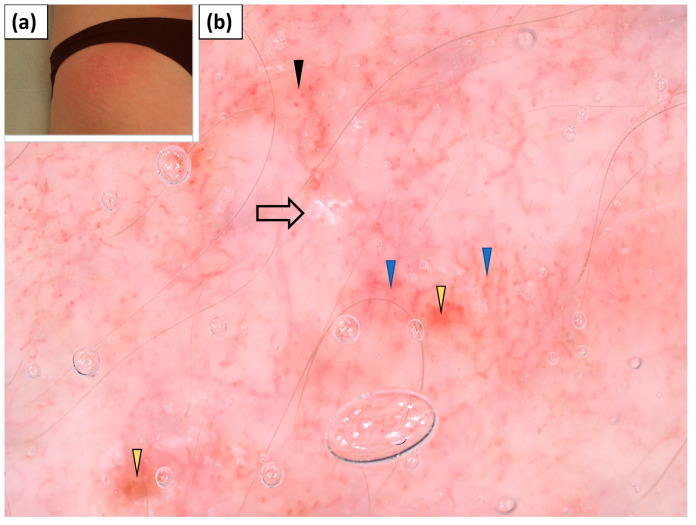
(**a**) Clinical presentation of holster sign. (**b**) Videodermoscopy of holster sign showed irregularly distributed dotted vessels (black arrowhead) and linear branched vessels with branches (blue arrowhead), lake-like structures (yellow arrowhead), and white patchy scales (black arrow).

Holster sign was present in one patient. Dotted and linear vessels with branches arranged in an unspecific pattern were noted. White scaling of a patchy distribution was also observed. In addition, single erosions were present (Table 4, Figure 9).

**Figure 10:**
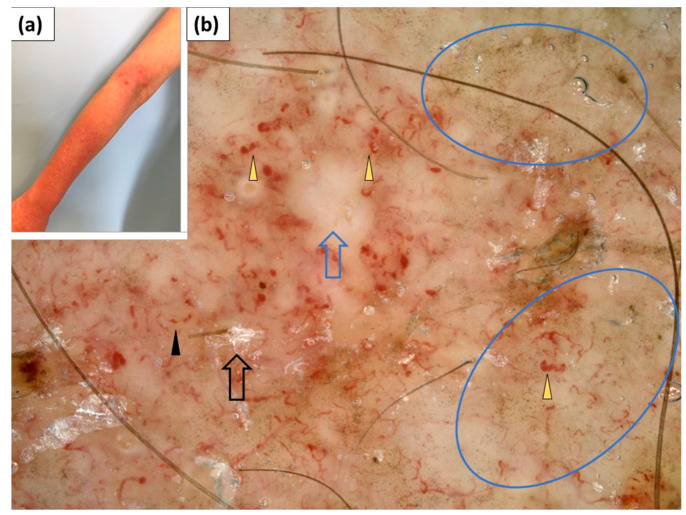
(**a**) Clinical presentation of erythematous lesions on the forearms. (**b**) Videodermoscopy of the lesions on the forearms showed irregularly distributed dotted vessels (black arrowhead) and linear curved vessels (yellow arrowhead), white patchy scales (black arrow), white structureless areas (blue arrow), and irregularly distributed brown-grey dots (blue circle).

In addition, four patients presented with erythematous and scaling lesions on the forearms. Polymorphic vessels of unspecific arrangement were present in all four cases—linear vessels (100%) were accompanied by dotted (75%), linear curved (50%), or linear vessels with branches (25%). White or yellow scaling of patchy distribution was present in 75% and 50% of cases, respectively. Pink structureless areas were present in two cases. In one case, white structureless areas were observed. In another case, yellow structureless areas were noted. Erosions were present in three patients, and the “sticky fiber” sign was observed under dermoscopy in a single case (Table 4, Figure 10).

## 4. Discussion

The (video)dermoscopic features of skin symptoms in the course of DM have not been extensively studied so far [7,8,9,10,11,12,13,14,15]. Data from the literature point at potential application of (video)dermoscopy in the assessment of nailfolds (capillaroscopy) [7,8,9,10], scalp (trichoscopy) [11,12], filiform papillae of the tongue [13], hand lesions [14], and cutaneous changes in erythrodermic DM [15].

Capillaroscopy use has great potential in DM, and several studies confirmed the application of handheld dermatoscopes and videodermatoscopes in the assessment of nailfold capillaries [7,8,9,10]. (Video)dermoscopy enables the visualization of “bushy” capillaries, dilated capillaries, nailfold bleedings, and avascular areas. The results of our videocapillaroscopic assessment are consistent with data from the literature and confirm the high prevalence of nailfold vessel abnormalities in patients with DM.

Trichoscopic findings in DM were initially reported by Jasso-Oliveres et al. [11] in 2017. The authors observed scalp involvement in 24 out of 31 (77.4%) patients with DM. The main clinical presentations included nonscarring alopecia (87.5%), erythema (83.3%), pruritus (70.8%), and poikiloderma (51.6%). Trichoscopy was performed in 28 patients and the most frequent findings were enlarged tortuous capillaries (71.2%), peripilar casts (57.1%), tufting, and interfollicular scales (50.0%). “Bushy” capillaries, peri- and interfollicular pigmentation, and vascular lake-like structures were observed in a smaller percentage of cases [11]. Interestingly, in the aforementioned study, the authors observed under trichoscopy dilated capillaries regardless of the clinical manifestations of scalp involvement.

It is also worth noting that the biggest study to date on the trichoscopic findings in DM was carried out in the Mexican population [11]. Little is known about the trichoscopic features of DM in Caucasians (Fitzpatrick skin phototype I–II).

Scalp lesions in the course of DM may be misdiagnosed as seborrheic dermatitis or psoriasis. Trichoscopy seems to be a useful non-invasive tool in differentiating these entities. Trichoscopy was also found to enhance the differential diagnosis of erythroderma [12,15]. Sławińska et al. [15] reported the presence of inter- and perifollicular pigmentation, venous lake-like structures, and perifollicular double white-pink concentric rings under trichoscopy in the erythrodermic variant of DM (n = 1). On the other hand, Golińska et al. [12] analyzed trichoscopic features in two patients with erythrodermic DM and observed white (50%) or yellow (50%) scales, branched (50%) or serpentine (100%) vessels, whitish-pinkish (50%) or orange (50%) structureless areas, and inter- (50%) or perifollicular (50%) brown pigmentation. In our study, we found a high prevalence of yellow dots (60%) and hair diameter diversity (60%) in patients with DM. However, these trichoscopic features are most probably related to coexisting androgenetic alopecia. Thin and thick linear vessels with branches (analogous to “bushy capillaries” of the nailfolds), as well as linear curved vessels (corresponding to tortuous vessels in capillaroscopy), were frequently observed. Therefore, more than half of the patients in our study showed polymorphous vessels under trichoscopy.

There are scarce data in the literature on the (video)dermoscopic features of other cutaneous manifestations of DM [14,15]. In a single case report, surface scaling, dotted vessels on a pink background and discrete pigmentations arranged in parallel ridge pattern were present under dermoscopy of “mechanic’s hand”. In addition, Gottron’s papules showed surface scales, dotted vessels on a pink background, and slightly brown structureless areas [14]. Sławińska et al. [15] reported the presence of linear vessels with branches and linear curved vessels of unspecific distribution, white-pinkish and brown structureless areas, as well as red irregular globules (lake-like structures) in one case of erythrodermic variant of amyopathic DM. We also observed a high prevalence of linear vessels, linear vessels with branches, and linear curved vessels, intermingled with white or pink structureless areas, in various cutaneous lesions in DM.

Data from the literature also suggest that (video)dermoscopy may be useful in the evaluation of filiform papillae of the tongue in connective tissue diseases (CTD) [13]; however, in our study, we did not perform videodermoscopy in this location.

It is worth noting that DM shows some dermoscopic similarities with lupus erythematosus (LE). In a recent study by Chanprapaph et al. [17] on trichoscopic findings in CTDs, a decrease in the hair shaft number, presence of white dots, brown honeycomb pattern, white patches, interfollicular scales, dotted vessels, and thin or thick arborizing vessels was observed with comparable frequency in DM and systemic lupus erythematosus (SLE). On the other hand, perifollicular red-brown pigmentation, perifollicular scaling, and tortuous dilated capillaries, referred to by the authors as “microaneurysmal blood vessels”, were found to be specific for DM, while hair shaft hypopigmentation was significantly more common in SLE. Under capillaroscopy, dilated capillaries, microhemorrhages, capillary dropouts, avascular areas, and tortuous capillaries may be observed in both DM and SLE. On the other hand, “bushy” capillaries are suggested to be specific for DM [18]. To the best of our knowledge, no direct comparison between the dermoscopic findings in cutaneous manifestations of DM and LE has been made so far. In cutaneous lupus erythematosus (CLE), follicular plugs, pink or white structureless areas, white patchy scaling and patchy dotted, linear, linear branched, and linear curved vessels may be observed with varying frequencies depending on the clinical variant of CLE [19]. Undoubtedly, further studies with side-by-side comparisons of the dermoscopic findings in cutaneous manifestations of DM and LE are needed.

The presence of vascular changes within the proximal nailfolds, visualized by capillaroscopy, is already a well-known issue in DM. Our study demonstrates that an abnormal vascular pattern, consisting of polymorphous vessels and structureless areas, may also be present under dermoscopy in cutaneous lesions, including Gottron’s papules and Gottron’s sign. In addition, abnormal vessels may also be observed on the scalp. Thick linear branched vessels and linear curved vessels under dermoscopy may correspond to dilated, tortuous, or “bushy” capillaries observed on capillaroscopy, and structureless areas may correspond to avascular areas present in the nailfolds. In addition, the unspecific vascular pattern on (video)dermoscopy may be considered analogous to a disorganized vessel architecture on capillaroscopy.

Dermoscopy may be of value in the evaluation of unspecific skin manifestations of DM, particularly in patients who will never develop muscle weakness (clinically amyopathic DM) or in patients referred to a dermatologist before the development of muscle weakness (praemyopathic DM).

To the best of our knowledge, this is the first study that presents detailed evaluation of (video)dermoscopic features of various cutaneous manifestations of DM. Further research is needed in order to reliably evaluate the applicability of non-invasive skin imaging methods in the differential diagnosis of DM.

Limitations of the current study include a small sample size, single-center design, inclusion of only Caucasian patients (Fitzpatrick skin phototype I–III), and lack of histopathological correlation.

## 5. Conclusions

Polymorphic vessels, including linear, linear branched, and linear curved vessels, intermingled with white or pink structureless areas, are commonly observed under dermoscopy in various skin manifestations of DM. Dermoscopy of the nailfolds (capillaroscopy), scalp (trichoscopy), and active skin lesions may be of value in the preliminary diagnosis of DM.

## Figures and Tables

**Table 1:** Clinical characteristics of study participants (SD–standard deviation).

Clinical Characteristics	
Gender, n (%)	
Male	5 (33.3)
female	10 (66.7)
Age, years	
Mean ± SD	48.7 ± 19.4
range	18–87
Time since first symptoms, months	
Mean ± SD	17.3 ± 26.0
range	1–96
Time since diagnosis, years	
Mean ± SD	1.13 ± 2.3
range	0–8
Muscle involvement, n (%)	9 (60.0)
Hair loss, n (%)	8 (53.3)
Associated malignancy, n (%)	1 (6.7)

**Table 2:** Dermoscopic features of the nailfold in patients with dermatomyositis (P1–P15—patient numbers).

Nailfold Capillaroscopic Findings	P1	P2	P3	P4	P5	P6	P7	P8	P9	P10	P11	P12	P13	P14	P15	Total (% of Cases with Abnormal Capillaroscopy/% of All Cases))
Tortuous vessels	-	-	+	+	+	+	-	-	-	-	+	+	+	+	-	8 (72.7/53.3)
“bushy” capillaries	-	-	-	+	+	-	-	-	-	-	+	-	+	+	-	5 (45.5/33.3)
Elongated capillaries	-	-	+	+	+	+	-	+	-	-	+	+	+	+	+	10 (90.9/66.7)
Dilated capillaries	-	-	+	+	+	+	-	-	-	-	+	+	+	+	-	8 (72.7/53.3)
Giant capillaries	-	-	-	-	+	-	-	-	-	-	+	-	+	+	-	4 (36.4/26.7)
Subpapillary plexus	-	-	-	+	+	+	-	-	-	-	+	+	+	+	+	8 (72.7/53.3)
Hemorrhages	+	-	-	+	+	+	-	+	-	-	+	-	+	-	+	8 (72.7/53.3)
Disorganized vessel architecture	-	-	+	+	+	+	-	-	-	-	+	+	+	+	+	9 (81.8/60.0)
Avascular areas	-	-	+	+	+	+	-	-	-	-	+	+	+	+	+	9 (81.8/60.0)

**Table 3:** Trichoscopic findings in patients with dermatomyositisi (P1–P15—patient numbers).

Trichoscopic Features	P1	P2	P3	P4	P5	P6	P7	P8	P9	P10	P11	P12	P13	P14	P15	Total (% of All Cases)
**Follicular openings**																
Absence of openings	-	-	-	+	+	+	+	-	+	-	-	-	-	-	-	5 (33.3)
White dots	-	-	-	-	-	-	+	-	-	-	-	-	-	-	-	1 (6.7)
Yellow dots	-	+	+	+	+	+	-	+	-	+	+	+	-	-	-	9 (60.0)
Red follicular dots	-	-	-	-	+	-	-	-	-	-	-	-	-	-	-	1 (6.7)
Dilated follicles	-	+	-	-	+	-	-	+	-	-	+	-	-	-	-	4 (26.7)
**Hair shafts**																
Hair diameter diversity	-	+	-	+	+	+	-	+	+	+	+	+	-	-	-	9 (60.0)
Pili torti	-	-	-	-	+	-	-	-	-	-	-	-	-	-	-	1 (6.7)
**Perifollicular surface**																
Scaling	-	+	-	-	-	-	+	-	-	-	-	-	+	-	-	3 (20.0)
Erythema	-	-	+	-	+	-	+	-	+	-	+	-	-	-	-	5 (33.3)
Pigmentation	-	-	+	-	-	+	+	-	+	-	+	+	-	-	-	6 (40.0)
White halo	-	-	-	-	-	+	-	-	-	-	-	-	-	-	-	1 (6.7)
**Interfollicular surface**																
White structureless areas	-	-	-	-	+	+	+	-	-	-	-	-	-	-	-	3 (20.0)
Pink structureless areas	-	-	-	+	-	+	-	-	-	-	-	-	-	-	-	2 (13.3)
White scales	-	-	-	-	+	-	-	-	-	-	-	-	+	-	+	3 (20.0)
Yellow scales	-	+	-	-	+	-	-	-	-	-	-	-	+	-	-	3 (20.0)
Honeycomb pigment pattern	-	-	-	-	-	+	+	+	+	-	+	+	-	-	-	6 (40.0)
Hemorrhages	-	+	-	-	-	-	-	-	-	-	-	-	-	-	-	1 (6.7)
Erosions	-	+	-	-	-	-	-	-	-	-	-	-	-	-	-	1 (6.7)
**Vessel morphology**																
Dotted	-	-	-	+	-	+	-	-	-	-	+	+	-	-	-	4 (26.7)
Linear	-	+	-	+	+	+	-	+	-	-	+	+	+	-	+	9 (60.0)
Thin linear branched	-	+	-	+	+	+	+	+	+	+	+	+	+	-	+	12 (80.0)
Thick linear branched	-	+	-	+	+	-	-	+	-	-	+	-	-	-	+	6 (40.0)
Linear curved	-	-	-	+	+	+	-	+	-	-	+	+	+	-	+	8 (53.3)
Polymorphous	-	-	-	+	+	+	-	+	-	-	+	+	+	-	+	8 (53.3)
Lake-like structures	-	-	-	-	+	-	-	-	-	-	-	+	-	-	-	2 (13.3)

**Table 4:** Dermoscopic features of the cutaneous lesions in dermatomyositis.

Dermoscopic Features	Gottron’s Papules n = 13	Gottron’s Sign (Elbows) n = 12	Gottron’s Sign (Knees) n = 5	Heliotrope n = 3	Face n = 7	V-Neck Sign n = 2	Holster Sign n = 1	Forearms n = 4
**Morphology of vessels, n (%)**								
Dotted	12 (92.3)	10 (83.3)	5 (100.0)	0 (0.0)	0 (0.0)	1 (50.0)	1 (100.0)	3 (75.0)
Linear	7 (53.9)	8 (66.7)	3 (60.0)	0 (0.0)	5 (71.4)	1 (50.0)	0 (0.0)	4 (100.0)
Linear with branches	6 (46.2)	5 (41.7)	3 (60.0)	3 (100.0)	5 (71.4)	2 (100.0)	1 (100.0)	1 (25.0)
Linear curved	4 (30.8)	3 (25.0)	0 (0.0)	0 (0.0)	3 (42.9)	2 (100.0)	0 (0.0)	2 (50.0)
Polymorphic	10 (76.9)	10 (83.3)	4 (80.0)	0 (0.0)	5 (71.4)	2 (100.0)	1 (100.0)	4 (100.0)
**Distribution of vessels, n (%)**								
Uniform	0 (0.0)	1 (8.3)	1 (20.0)	2 (66.7)	2 (28.6)	0 (0.0)	0 (0.0)	0 (0.0)
Clustered	0 (0.0)	2 (16.7)	0 (0.0)	0 (0.0)	1 (14.3)	1 (50.0)	0 (0.0)	0 (0.0)
Peripheral	0 (0.0)	0 (0.0)	0 (0.0)	0 (0.0)	0 (0.0)	0 (0.0)	0 (0.0)	0 (0.0)
Unspecific	13 (100.0)	9 (75.0)	4 (80.0)	1 (33.3)	4 (57.1)	2 (100.0)	1 (100.0)	4 (100.0)
**Color of scales, n (%)**								
White	10 (76.9)	10 (83.3)	4 (80.0)	1 (33.3)	1 (14.3)	2 (100.0)	1 (100.0)	3 (75.0)
Yellow	1 (7.7)	0 (0.0)	0 (0.0)	0 (0.0)	1 (14.3)	0 (0.0)	0 (0.0)	2 (50.0)
**Distribution of scales, n (%)**								
Diffuse	1 (7.7)	0 (0.0)	1 (20.0)	0 (0.0)	1 (14.3)	0 (0.0)	0 (0.0)	0 (0.0)
Central	0 (0.0)	0 (0.0)	0 (0.0)	0 (0.0)	0 (0.0)	0 (0.0)	0 (0.0)	0 (0.0)
Peripheral	0 (0.0)	2 (16.7)	0 (0.0)	0 (0.0)	0 (0.0)	0 (0.0)	0 (0.0)	0 (0.0)
Patchy	9 (69.2)	9 (8.3)	3 (60.0)	1 (33.3)	1 (14.3)	2 (100.0)	1 (100.0)	4 (100.0)
**Follicular findings, n (%)**								
Follicular plugs	5 (38.5)	1 (8.3)	2 (40.0)	0 (0.0)	2 (28.6)	0 (0.0)	0 (0.0)	0 (0.0)
Follicular red dots	0 (0.0)	0 (0.0)	0 (0.0)	1 (33.3)	0 (0.0)	0 (0.0)	0 (0.0)	0 (0.0)
Perifollicular pigmentation	0 (0.0)	0 (0.0)	0 (0.0)	0 (0.0)	1 (14.3)	0 (0.0)	0 (0.0)	0 (0.0)
**Morphologies/colors, n (%)**								
White structureless areas	6 (46.2)	5 (41.7)	2 (40.0)	0 (0.0)	1 (14.3)	0 (0.0)	0 (0.0)	1 (25.0)
Pink structureless areas	10 (76.9)	4 (33.3)	3 (60.0)	0 (0.0)	1 (14.3)	0 (0.0)	0 (0.0)	2 (50.0)
Yellow structureless areas	0 (0.0)	0 (0.0)	0 (0.0)	0 (0.0)	1 (14.3)	0 (0.0)	0 (0.0)	1 (25.0)
Red dots/globules	5 (38.5)	2 (16.7)	1 (20.0)	0 (0.0)	0 (0.0)	1 (100.0)	0 (0.0)	1 (25.0)
Brown globules	0 (0.0)	0 (0.0)	1 (20.0)	0 (0.0)	0 (0.0)	0 (0.0)	0 (0.0)	0 (0.0)
Yellow globules	0 (0.0)	0 (0.0)	0 (0.0)	3 (100.0)	0 (0.0)	0 (0.0)	0 (0.0)	0 (0.0)
Grey dots	0 (0.0)	0 (0.0)	0 (0.0)	1 (33.3)	0 (0.0)	0 (0.0)	0 (0.0)	1 (25.0)
**Specific clues, n (%)**								
Stellate brown structures	1 (7.7)	1 (8.3)	0 (0.0)	0 (0.0)	1 (14.3)	0 (0.0)	0 (0.0)	0 (0.0)
Speckled brown pigmentation	0 (0.0)	3 (25.0)	0 (0.0)	0 (0.0)	0 (0.0)	0 (0.0)	0 (0.0)	0 (0.0)
Yellowish crust	0 (0.0)	0 (0.0)	1 (20.0)	0 (0.0)	0 (0.0)	0 (0.0)	0 (0.0)	0 (0.0)
Erosion	0 (0.0)	0 (0.0)	1 (20.0)	0 (0.0)	1 (14.3)	0 (0.0)	0 (0.0)	3 (75.0)
“sticky fiber” sign	0 (0.0)	0 (0.0)	0 (0.0)	0 (0.0)	1 (14.3)	0 (0.0)	0 (0.0)	1 (25.0)

## Data Availability

Data are available from corresponding author upon request.
